# RETRACTED: Kebede et al. Determinants of Disease Progression in Autosomal Dominant Polycystic Kidney Disease. *J. Pers. Med.* 2024, *14*, 936

**DOI:** 10.3390/jpm16010046

**Published:** 2026-01-09

**Authors:** Molla Asnake Kebede, Yewondwosen Tadesse Mengistu, Biruk Yacob Loge, Misikr Alemu Eshetu, Erkihun Pawlos Shash, Amenu Tolera Wirtu, Jickssa Mulissa Gemechu

**Affiliations:** 1 Department of Internal Medicine, School of Medicine, College of Medicine and Health Science, Mizan-Tepi University, Mizan-Aman P.O. Box 260, Ethiopia; mollaasnake@mtu.edu.et (M.A.K.); misikralemu@mtu.edu.et (M.A.E.); erkyihunpawlos@mtu.edu.et (E.P.S.); 2 Department of Nephrology, College of Health Sciences, Addis Ababa University, Addis Ababa P.O. Box 9086, Ethiopia; yewonst@yahoo.com; 3 Durame General Hospital, Internal Medicine Unite, SNNPR, Durame P.O. Box 143, Ethiopia; cescfan.biru@yahoo.com; 4 Meritus Medical Center, Meritus School of Osteopathic Medicine, Hagerstown, MD 21742, USA; wirtuat@ul.edu.lr; 5 Department of Foundational Medical Studies, Oakland University William Beaumont School of Medicine, Rochester, MI 48309, USA

The journal retracts the article entitled “Determinants of Disease Progression in Autosomal Dominant Polycystic Kidney Disease“ [1], cited above.

Following publication, two of the authors contacted the Editorial office to raise concerns regarding the ownership of the data presented in this article [1].

Adhering to our standard procedure, an investigation was conducted by the Editorial Office and the Editorial Board that confirmed that the underlying data was re-published from a doctoral thesis [2], produced by a co-author, without citation or the securing of appropriate institutional permission. Further concerns relating to the validity and origins of Figures 1–4, could not be adequately resolved. As a result, the Editorial Board decided to retract this article as per MDPI’s retraction policy (https://www.mdpi.com/ethics#_bookmark30).

This retraction was approved by the Editor-in-Chief of *Journal of Personalized Medicine*.

Authors Yewondwosen Tadesse Mengistu, Biruk Yacob Loge and Erkihun Pawlos Shash agreed to this retraction. The remaining authors did not provide a comment on this decision.
